# Enhanced saccharification of lignocellulosic agricultural biomass and increased bioethanol titre using acclimated *Clostridium thermocellum* DSM1313

**DOI:** 10.1007/s13205-017-0606-z

**Published:** 2017-04-13

**Authors:** M. Nisha, K. Saranyah, Mukund Shankar, L. M. Saleena

**Affiliations:** 1 Department of Biotechnology, School of Bioengineering, SRM University, Kattankulathur, Chennai, Tamil Nadu 603203 India; 2 Department of Chemical Engineering, School of Bioengineering, SRM University, Kattankulathur, Chennai, Tamil Nadu 603203 India

**Keywords:** *Clostridium thermocellum*, Acclimation, Lignocellulose, Cellobiose, Reducing sugars, Ethanol

## Abstract

Consolidated bioprocess assures an efficient lignocellulosic conversion to fermentable sugars and subsequently to bioethanol. Such a single-step hydrolysis and anaerobic fermentation was achieved with acclimated *Clostridium thermocellum* DSM 1313 on different mildly pre-treated agricultural lignocellulosic residues without any additional enzymes/and strains. Acclimation was achieved by serially sub-culturing in increasing concentration of individual substrates, such as rice husk, sugarcane bagasse, and banana pseudostem in the standard media, with cellobiose as an adjunct. The acclimated cellulolytic thermophile exhibited an early log phase entry with enhanced growth compared to the direct inoculation experiments with unacclimated culture. Around 672 mg/g of reducing sugar was produced from sugarcane bagasse media and 636 mg/g from rice husk media and 513 mg/g from banana pseudostem media with the acclimated organism. Bioethanol production also doubled in experiments with serially acclimated cultures, with a maximum of 1.21 and 1.0 g/L ethanol titre from sugarcane bagasse and rice husk, respectively. The serial acclimation experiments have increased the saccharification potentials of the organism towards the respective lignocellulosic substrates and also enhanced the bioethanol production.

## Introduction

The first-generation biofuels have promoted edible crops as feedstocks for bioethanol production. The seasonal, regional, and rate of growth variations of the edible feed stocks have been reported to lead inconsistent supply for bioethanol production (Kim and Dale 2004). Today, second-generation biofuels are considered as advanced biofuels, since the feedstock generally used is not food crops, such as in first-generation biofuels. Various methods, such as physico-chemical/and enzymatic hydrolytic techniques, have been used for converting lignocellulosic composite to reducing sugars for bioethanol production. The widely adopted pre-treatments for lignocellulosic substrates (LCSs), such as steam explosion, acid hydrolysis, and ammonia fibre expansion, are often expensive and limit commercialization. In addition, in most cases, pre-treatments generate toxic substances necessitating a detoxification stage before hydrolysis (Maki et al. 2009; Brodeur et al. 2011).

Microbial hydrolysis is a promising approach that reduces time and cost in LCS hydrolysis in comparison with the non-microbial processing techniques. Only a few known microorganisms have the capability to degrade and hydrolyse recalcitrant plant biomass. Cellulolytic thermophiles are increasingly researched due to the high tolerance level to fluctuating pH, temperature, and environmental changes. Their enzymes take in a crucial role in industries due to its thermo-stability (Pandey 2011). *Clostridium thermocellum* is an anaerobic, spore-forming cellulolytic thermophile producing multi-enzyme complex consisting of various hydrolytic enzymes as well as extracellular individual free enzymes. Having both these enzyme systems is a rare characteristic in the microbial world due to which *C. thermocellum* is an efficient biomass degrader along with ethanolgenic capabilities (Wilson 2011; Paye et al. 2016). Application of *C. thermocellum* simplifies the LCS bioprocess by simultaneous hydrolysis and fermentation of the hydrolysate within the same pot. The consolidated process involves least investment in enzyme production, cellulose hydrolysis, and enzyme recovery. However, irrespective of the technological advancements, the technical and economic constrains of converting the cellulosic material into fermentable sugars for the production of biofuels remain unsolved.

Researches are being carried out to enhance the hydrolytic capability of the wild strain *C. thermocellum*. *C. thermocellum* with superior hydrolytic capabilities is, however, reported to produce low-level cellulosomes (You et al. 2012). Even though the thermophile is claimed to completely convert pure cellulose to ethanol, it does not produce similar results with other cellulosic substrates (Pandey 2011). This work deduces the effect of microbial hydrolysis of *C. thermocellum* after acclimation towards LCSs. The efficiency of microbial hydrolysis was established in terms of released reducing sugars yield, recovery of residual LCS, and final ethanol concentration. Several mildly pre-treated agricultural residues were microbially hydrolysed using wild and acclimated *C. thermocellum* DSM1313 without any additional enzyme or chemical supplements.

## Materials and methods

### Lignocellulosic substrates: collection and processing

Rice husks (*Oryza sativa* L.), banana pseudostem (*Musa paradisiaca* L.), were collected after harvest from Chunambedu, Kanchipuram district, Tamil Nadu, India. Sugarcane bagasse (*Saccharum officinarum* L.) was collected from Pavunjur, Kanchipuram district, Tamil Nadu, India. Collected samples were air-dried, chopped into fine pieces with kitchen aid mixer, and sifted through BS-410, mesh 36. Ground LCSs were then stored in zipper lock polypropylene plastic bags at room temperature until analysis and treatment.

### LCSs: preparation and pre-treatment

Rice husk (RH), sugarcane bagasse (SB), and banana pseudostem (BP) were washed with water and dried overnight at 50 °C. Dried samples were pre-treated with 250 mM NaOH solution (Kiyoshi et al. 2015) for 20 min at 121 °C. Treated LCSs were neutralised with repeated distilled water wash and dried in 50 °C (overnight) and cooled in desiccator until constant weight was achieved.

### Field emission scanning electron microscope analysis

Field emission scanning electron microscope (FESEM) samples were non-conductive, and the mode was low and extended low vacuum. The samples were spread on the carbon tape and analysed with an accelerating voltage of 20 kV using FEI’s Quanta 200 FEG.

### Fourier transform infrared spectroscopy analysis

Samples were ground along with potassium bromide in the ratio 1:10 and pelleted under pressure to form uniformly spread thin discs ~10 mm diameter and analysed by Fourier transform infrared spectroscopy (FTIR) (Cary 660; Agilent Technologies). Spectra were obtained by averaging 16 scans from 4000 to 400 cm^−1^ at 4 cm^−1^ resolution. The blank scan was an average of 64.

### Colorimetric determination of cellulose

The sample (500 mg) was homogenized with distilled water to which 5 mL acetic acid—nitric acid reagent was added and placed in a boiling water bath for 30 min. Cooled sample was centrifuged (4250*g* for 5 min), and the pellets were washed with distilled water followed by the slow addition of 10 mL of 67% H_2_SO_4_ (v/v) and mixed intermittently. This sample was kept aside for 1 h and diluted to 100 mL and centrifuged (1500*g* for 10 min) if any precipitate or turbidity present. For the assay, 0.1 mL of diluted sample was taken and 4.9 mL distilled water was added. 10 mL ice cold anthrone reagent (0.2 g in 100 mL conc. H_2_SO_4_) was added keeping the tube in an ice bath. It was then kept in boiling water bath for 15 min, cooled rapidly in ice bath. The absorbance of the blue green colour developed was read at 630 nm against a reagent blank (Updegraff 1969).

### Culture and storage of strain

*Clostridium thermocellum* DSM1313 was purchased from Leibniz Institute DSMZ-German Collection of Microorganisms and Cell Cultures. Stock cultures were maintained in DSM 122 media with 5 g/L of cellobiose as the carbon source. Long-term culture storage was done by re-suspending the pelleted cultures in DSM122 cellulose (10 g/L) media and 50% v/v glycerol at −80 °C.

### Inoculum preparation

All aseptic transfers pertaining to anaerobic cultivation were performed in an anaerobic hood flushed with nitrogen gas. 10% (v/v) of DSM122 cellulose medium (passaged for three batches) was the initial seed considered in all experiments unless otherwise mentioned.

### Microbial hydrolysis and fermentation of agricultural substrates using *Clostridium thermocellum*

#### Experimental setup I: direct inoculation

**Fig. 1:**
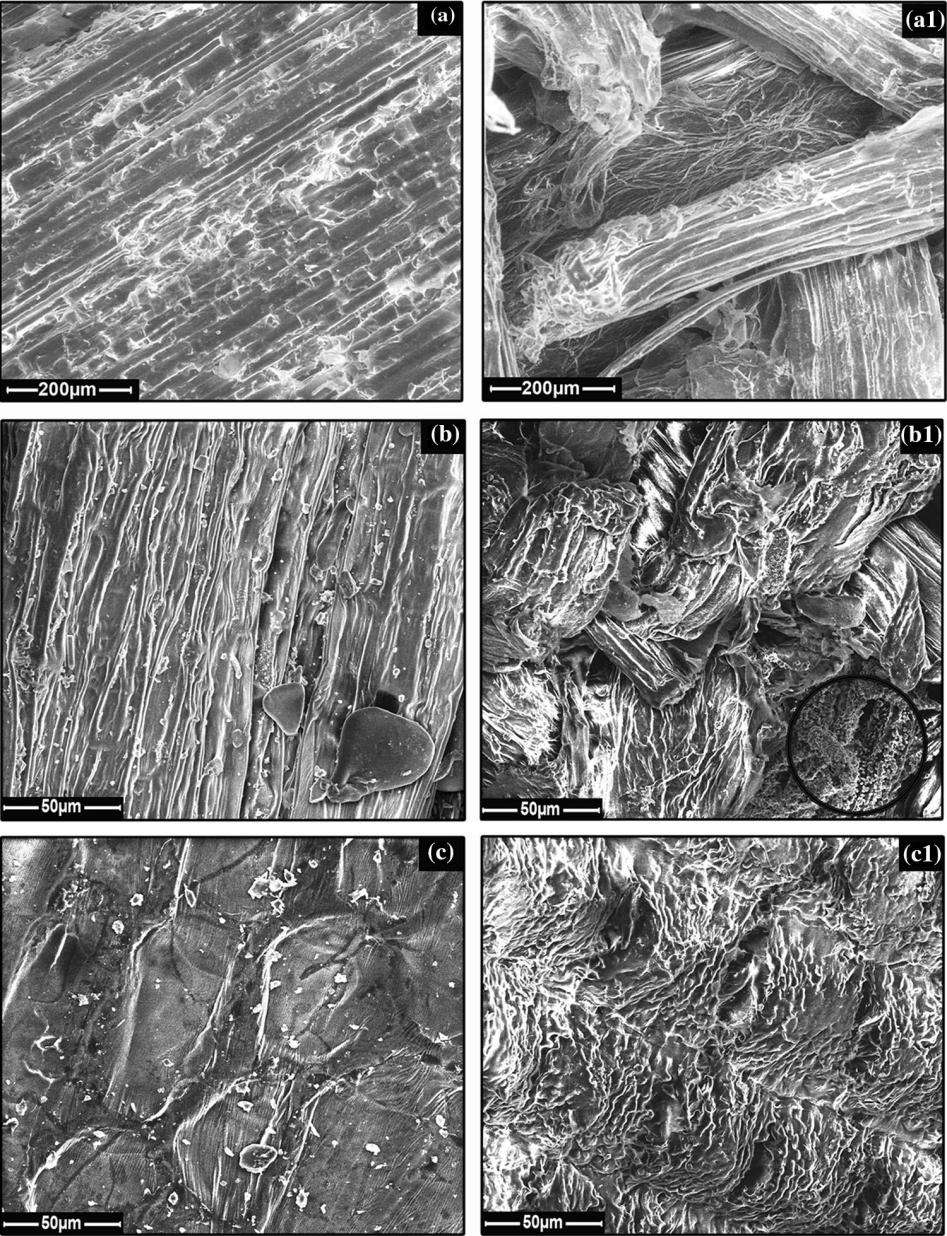
FESEM micrographs. **a** Untreated SB, **a1** treated SB, detached fibrils are depicted in the *circle*. **b** Untreated BP, **b1** treated BP, lignin particles attached to the fibres depicted in the *circle*. **c** Untreated RH, **c1** treated RH. Magnification *scale bar* is illustrated in each image

Cellulose ingredient in DSM 122 media was substituted by LCS under study. They were weighed and added based on their respective cellulose content gram (g) equivalent to recommended DSM cellulose concentration. 100 mL vials having 70 mL of the media were inoculated with 7 mL *C. thermocellum* seed inoculum. All vials were flushed with nitrogen gas and sealed with butyl rubber stoppers and aluminium crimps. Concurrently, all the vials were incubated for 120 h at 60 °C with an initial pH of 7.4. Reducing sugars and growth were recorded every 24 h. DSM 122 cellulose medium was the control. Triplicates were kept for all batches (Fig. 1).

#### Experimental setup II: serial acclimation experiment

Four different serial acclimation experiment (SAE) series viz, SAE1, SAE2, SAE3, and SAE4 were formulated based on the varying (g) equivalent quantity of LCS cellulose. Cellobiose was added as an adjunct to suffice the 10 g/L of total carbohydrate content as recommended in DSM 122 media composition. The whole experimental setup was carried out by serially sub-culturing from SAE1 to SAE4.

Formulation of the series was:

1. SAE1: 7.5 g/L of cellobiose and LCS (g) equivalent to 2.5 g/L of pure cellulose;
2. SAE2: 5.0 g/L of cellobiose and LCS (g) equivalent to 5.0 g/L of pure cellulose;
3. SAE3: 2.5 g/L of cellobiose and LCS (g) equivalent to 7.5 g/L of pure cellulose;
4. SAE4: LCS (g) equivalent to 10.0 g/L of pure cellulose.

Here, uniform volume of 70 mL in 100 mL serum vials was maintained for all trials. Inoculum for SAE1 and experimental setup1 was similar. Strategy involved a serial passaging of 96 h old SAE1 to SAE2 medium with 10% (v/v) inoculum. This inoculated medium after 96 h of incubation then served as a seed (10% v/v) for the next higher LCS concentration containing medium and so forth (SAE1 » SAE4). For every setup, the growth and reducing sugar concentrations were intermittently checked for every 24 h. Triplicates were kept for all batches.

#### Growth analysis

Grown cultures were vigorously mixed on a vortex mixer (at room temperature) to free the adhered cells from LCS, and the particles were allowed to settle for an hour (Fleming and Quinn 1971). Growth spectrometrically measured at 600 nm.

#### Colorimetric determination of reducing sugar

Reducing sugar analysis of all samples was conducted by 3,5-dinitrosalicylic acid assay (Miller 1959). 171 µL of DNS reagent was added to 9 µL sample supernatant (centrifuged at 9000*g*, 10 min) in a 96 well non-skirted PCR plate. The reaction mix was heated to 95 °C for 5 min in a thermocycler and subsequently held at 20 °C for 10 min for cooling and read at 540 nm. Total reducing sugar concentration was calculated from linear regression of the pre-assayed standard glucose and expressed as g/L.

### Data analysis and interpretation of the experimental setup I and II

#### Fraction values (*f*.RS)

To evaluate the hydrolysate based on release of reducing sugar, fraction values (*f*.RS) of the total reducing sugars (g/L) of every 24 h from a 5 day batch was calculated:

$$ f.{\text{RS}} = \frac{\text{total reducing sugar of nth hour}}{{{\text{total reducing sugar at }}0{\text{th hour}}}}. $$

Total reducing sugars in the broth when inferred as a fraction indicates consumption or release of sugars into the media at a particular time of analysis. *f*.RS values >1.0 signify release of reducing sugars, and *f*.RS values <1.0 signify consumption of the available, reducing sugars at that particular period. The rate of consumption will be equal to the rate of release when *f*.RS = 1 under a normal cell growth pattern (Fig. 3b).

#### Determination of residual LCS

The remnant LCS feed in the broth after fermentation was pelleted at 5000*g* for 15 min. The pellets were washed with double distilled water twice and filtered. The residuals were dried and cooled in a desiccator until a constant weight was achieved for gravimetric analysis (Dharmagadda et al. 2010).

### Chromatographic analysis

#### Identification of the mono-sugars from the derived hydrolysate

HPLC (UFLC LC-20 AD; Shimadzu RID-10A detector) analyses were performed to identify mono-sugars released at the end of 5 day microbial hydrolysis. Luna NH2 100A column having a length of 150 mm, ID of 4.6 mm, and pore size of 5 µm was considered. 80% acetonitrile in water was used as mobile phase with a flow rate adjustment to 3 mL/min and temperature at 30 °C (Lopez Hernandez et al. 1998). 10 µL of sample was injected. Glucose, galactose, arabinose, mannose, xylose, fructose (Supelco Monosaccharides kit), and cellobiose were the major standards used for the study.

#### Ethanol concentration

Ethanol concentration was analysed using reversed phase HPLC. Analyses were performed in HPLC 1260 infinity (Agilent Technologies) with equipped with G1329B autosampler. Water was used as a mobile phase in a 4.6 × 250 mm Zorbax Eclipse Plus C18 column with 5 µm pore size. 4% acetone was added to the mobile phase as a UV absorbing agent. Ethanol was detected as a negative peak. The intensity was measured with a PDA-UV detector at 235 nm. Samples were prepared by centrifuging the broth; 50 g of supernatant was distilled (79 ± 1 °C) to collect a uniform weight of 10 g. 10 µL of which was subjected to HPLC. The values were calculated from linear regression of the standards (Nisha et al. 2016).

## Results and discussion

This study explains the strategy of developing hydrolysate rich in mono-sugars from LCS for consequent ethanol production using *Clostridium thermocellum*. Pre-treatments, such as a steam explosion, acid/enzyme, or in combinations, are reported for lignin removal as well as for saccharification of LCS. Since *C. thermocellum* has feruloyl esterases which can release lignin from hemicellulose (Blum et al. 2000) and also due to the presence of a well-defined cellulolytic complex comprising of 72 different proteins along with 25 free enzymes (Taylor et al. 2009), only mild pre-treatments with dilute NaOH were considered to process the LCSs just enough to expose the cellulosic moieties.

### Pre-treatment of LCS

To develop robust microbes for efficient biomass conversion and bioethanol production, the quality of the pre-treated biomass is of prior importance. Naturally occurring biomass has a well-structured tightly arranged heterogeneous biochemical matrix with small-sized pores making it inaccessible to hydrolytic enzymes to act upon glycosidic bonds. This necessitates a pre-treatment before to hydrolysis to increase the porosity, hemicellulose solubilisation, lignin solubilisation, and lignin redistribution (Chaturvedi and Verma 2013). A physical process, such as grinding, before alkali pre-treatment is a necessity for increasing the surface area for microbial adherence and to decrease the cellulose crystallinity (Taherzadeh and Karimi 2008). Such physical parameters are a major contributor in improvising the conversion of biomass residues to fermentable sugars. Here, the finely ground substrates were sieved to ensure uniform granularity of maximum size of ~400 µm. Under study, the highest cellulose content was estimated in BP (53.7 ± 1.7%) followed by SB (42.3 ± 1.4%) and RH (26.2 ± 1.34%). LCSs were pre-treated with 250 mM of NaOH for the selective disruption of lignin with less carbohydrate degradation and minimal inhibitor formations (Kumar et al. 2009). Wang et al. (2010) reported that 1% NaOH pre-treatment for 30 min at 121 °C was sufficient to achieve a maximum lignin removal and gave significantly higher total reducing sugars than that with 3% NaOH (Wang et al. 2010).

### FESEM and FTIR analysis of the pre-treated substrates

The uniformity and the highly ordered patterns throughout the fibre length of all the substrates were lost. The inner fibres of the treated SB were exposed to the outer surface which possibly enhanced the accessible area for microbial adherence. The uniformly arranged fibres aligned in parallel to the axis bundle were disturbed in treated BP samples. Detached lignin particles were apparently visible in the distorted BP samples. Untreated RH has a highly ordered arrangement of fibrils giving a very smooth appearance, whereas treated RH had a complete distortion sufficient enough to expose the inner regions (Fig. 1).

**Fig. 2:**
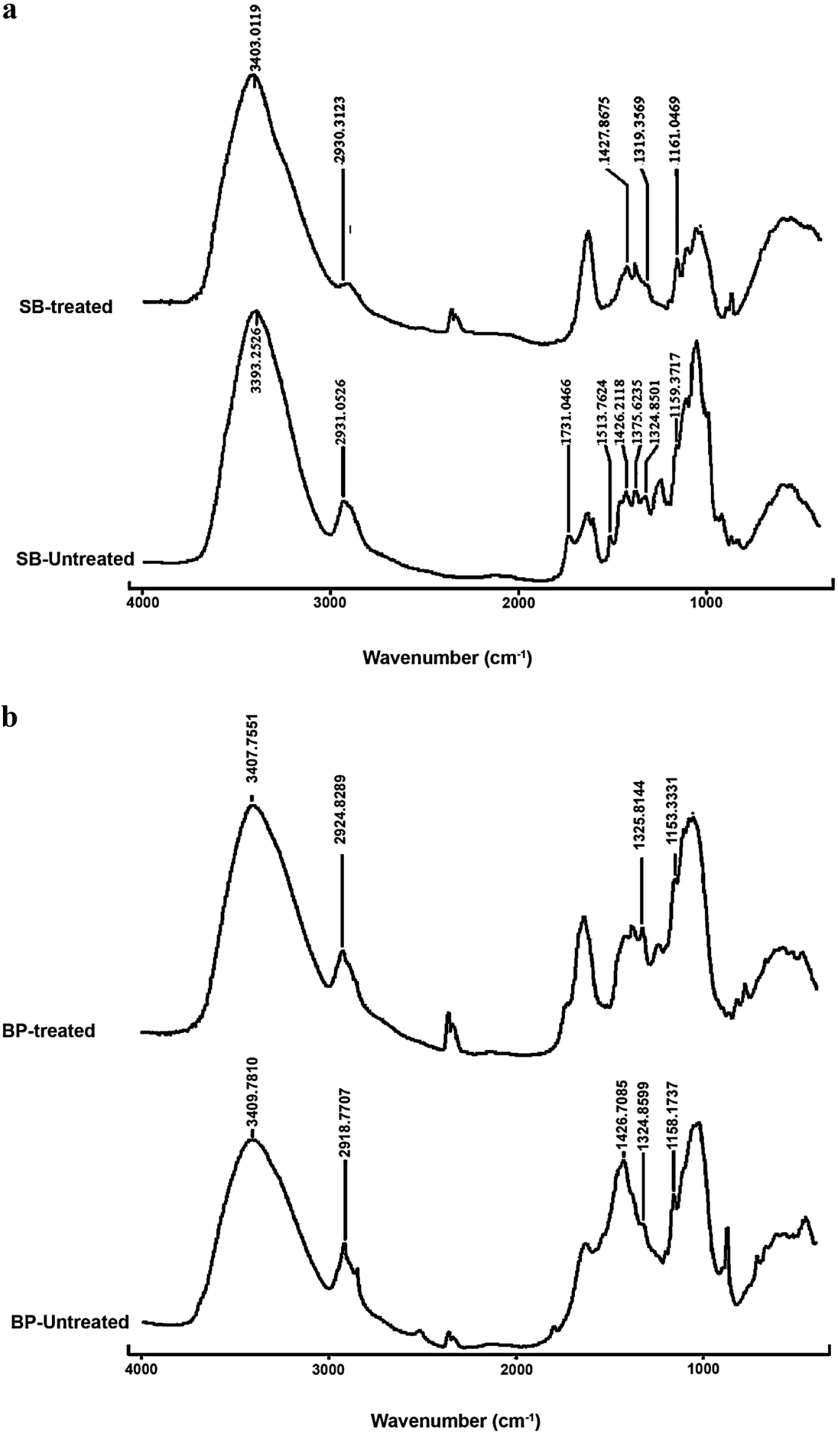

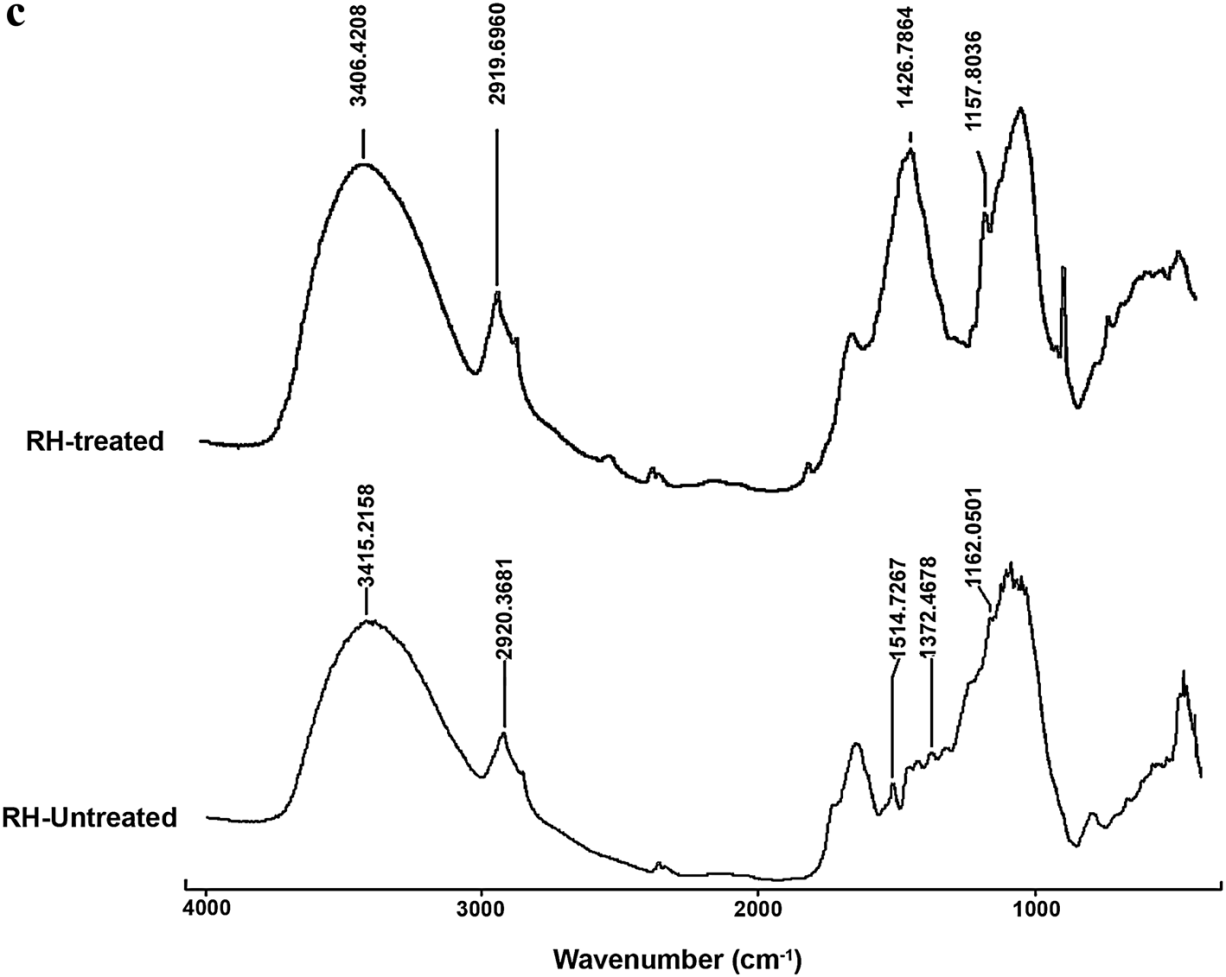
**a** FTIR spectrum of untreated and treated SB. **b** FTIR spectrum of untreated and treated BP. **c** FTIR spectrum of untreated and treated RH

To identify the changes occurred in functional groups of the lignin cellulose moieties of the pre-treated biomass were analysed in FTIR. All the major peak ranges considered for this study are mentioned in Table 1. Less absorbance at peak ‘a’ of treated SB infers the disruption of H bond of cellulose hydroxyl group (Sindhu et al. 2010). Similar effects were also seen in fingerprints specific to lignin linkages (Table 2; Fig. 2a). In treated BP, peaks showed an overall drop in intensities. Noticeable dip in peak ‘g’ is due to the bond disruption in glycoside linkage and aromatic groups of lignin (Guimarães et al. 2009). However, compared to other substrates, the decrease in bond intensity was not substantial (Table 2; Fig. 2b). The authors have not come across reports on delignification studies of RH with various alkaline pre-treatments. In treated RH, the peaks of ‘f’ were not detected and ‘b’, ‘g’, and ‘a’ showed reduced absorbance. One of the primary functional group related to lignin at peak ‘f’ linking the hemicellulose has disappeared (Ndazi et al. 2007; Luduena et al. 2011) (Table 2; Fig. 2c). Among the three substrates, better performance was observed in SB and RH.

**Table 1:** Assignment of major functional groups unique for lignin and cellulose in lignocellulosic substrates

Absorption band location (cm^−1^)	Assignment	Peak labels	References
3600–3100	H bond of OH group of cellulose	a	Pereira et al. (2014)
2937–2918	–CH stretch of methyl and methylene groups of cellulose	b	Pereira et al. (2014)
1731–1720	C=O stretch of ketone/aldehyde	c	Bilba et al. (2007)
1428–1425	Aromatic ring vibrations of lignin	e	Bilba et al. (2007)
1375–1320	Phenol hydroxyl stretch; lignin	f	Bilba et al. (2007)
1167–1151	Ester bond stretch of lignin and carbohydrate	g	Bilba et al. (2007)

**Table 2:** FTIR spectroscopic results of untreated and treated SB, BP, and RH

Peak label	SB				BP				RH				
	Untreated		Treated		Untreated		Treated		Untreated		Treated		
	WN	H	WN	H	WN	H	WN	H	WN	H	WN	H	
a	3393.3	1.505	3403.0	0.690	3409.8	1.864	3407.8	1.022	3415.2	2.605	3406.4		1.316
b	2931.1	0.645	2930.3	−0.061	2918.8	1.207	2924.8	0.525	2920.4	1.844	2919.7		0.963
c	1731.0	0.488	x		x		x		1514.7	1.501	x		
e	1426.2	0.684	1427.9	0.043	1426.7	1.737	x		x		x		
f	1375.6	0.687	1319.4	−0.162	x		x		1372.5	1.712	x		
	1324.9	0.664	x		1324.9	1.330	1325.8	0.602	x		x		
g	1159.4	1.188	1161.0	0.069	1158.2	1.517	1153.3	0.767	1162.1	2.624	1157.8		1.183

*WN* peak at (cm^−1^), *H* peak height (a.u.), *x* peak not detected

### *Clostridium thermocellum* and its behavioural growth trends in DSM122 cellulose media and DSM122 cellobiose media

**Fig. 3:**
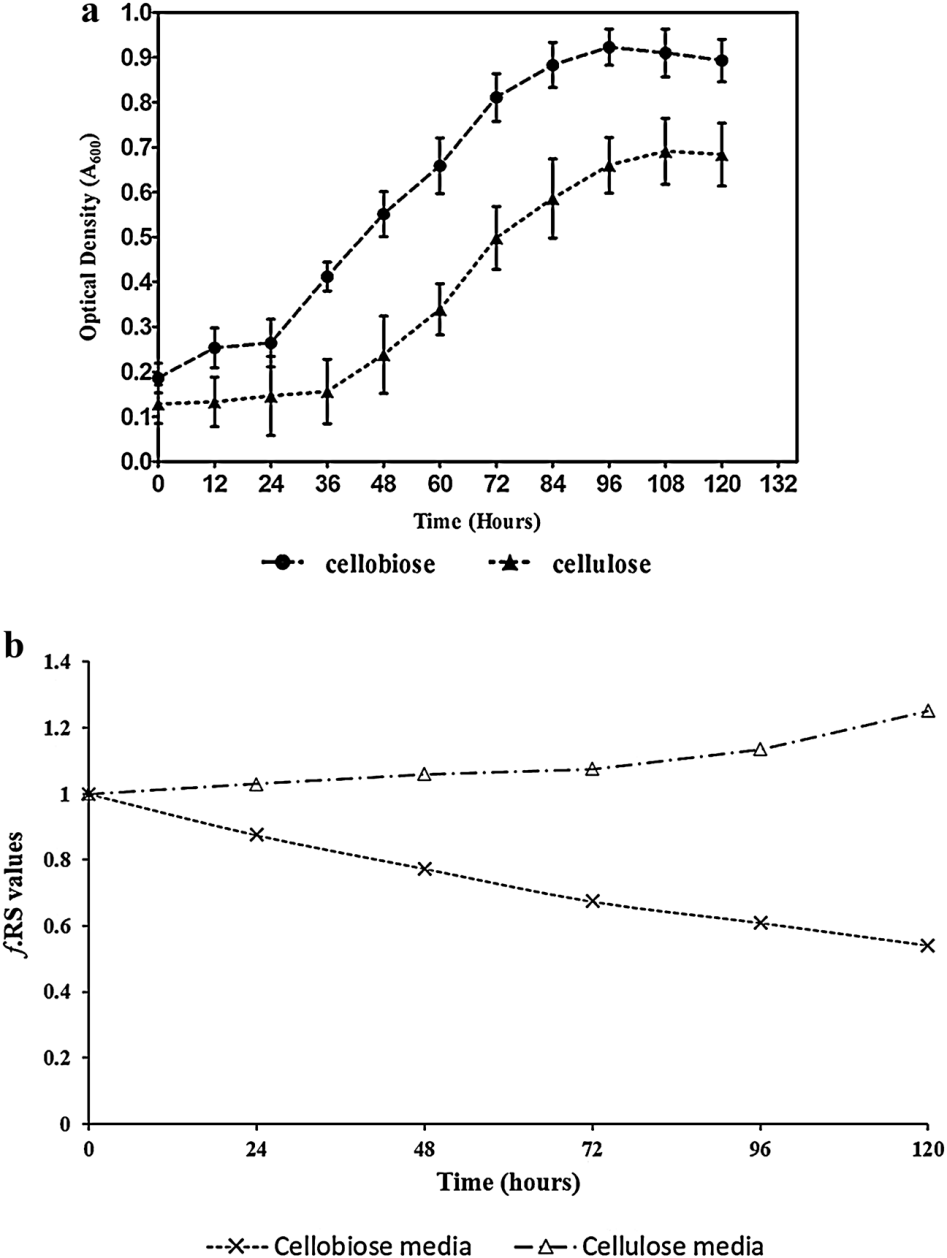
**a** Different growth pattern of *Clostridium thermocellum* in the standard cellulose and cellobiose media. In cellobiose media organism depicted an early catch up and increased OD_600_ values compared to cellulose media. However, the growth pattern was similar in both media. **b** Graphical representation of calculated *f*.RS from *Clostridium thermocellum* inoculated in cellobiose and cellulose medium. *f*.RS values from cellobiose media was <1, indicating its consumption by the organism and in cellulose media the *f*.RS value was >1 and increased each day indicating the release of reducing sugar due to hydrolysis exceeding the consumption rate of the organism

The growth of *C. thermocellum* in the standard cellulose and cellobiose media was observed to be similar (Fig. 3a) with a gradual pH shift to acidic range. The final pH values were 5.3 and 5.7 in cellobiose and cellulose media, respectively. The increased growth in these standard media infers the substrate familiarity and also its selective nature of action on the respective substrates. Even though the same growing seed culture was inoculated into the aforementioned standard substrates, 1.6× reduction in the total reducing sugar content was observed in cellobiose media, whereas an increase of 1.25× from the initial reducing sugar content was observed in the cellulose media. The 120th hour *f*.RS values were found to be 0.541 indicating consumption of reducing sugars and a fraction of 1.25 release of reducing sugars in the respective standard media (Fig. 3b). 64% of cellulose conversion was observed in cellulose media. Final ethanol concentration estimated was 0.54 and 1.14 g/L for cellobiose and cellulose media, respectively, which were in concurrent to published data (Weimer and Zeikus 1977; Tripathi et al. 2010).

### Microbial hydrolysis and anaerobic fermentation

#### Experimental setup I: direct Inoculation

In DSM122 cellulose media, the lag phase extended up to 36 h and attained a maximum OD_600_ within 120 h batch. However, for RH, SB, and BP, the growth of *Clostridium thermocellum* had lag phase extending up to 72, 76, and 84 h, respectively. Slow growth depicts the reduced competency of *Clostridium thermocellum* in adapting to new lignocellulose substrate, although the seed culture was vibrantly growing in cellulose. Similar observation was described elsewhere when active cultures grown in avicel were inoculated into pre-treated wood (Lynd 1989). The pH shift across 120 h had an acidic trend similar to the DSM122 cellulose media. According to Hörmeyer et al. (1988), the degradation of pre-treated LCSs was found to be equally effective in non-pH-controlled shake flask, in in vitro experiments with cell-free culture supernatant and also in vivo (cellulolyses) with pH regulation in a laboratory fermenter (Hörmeyer et al. 1988).

Soluble sugars keep accumulating in the media cultured with any cellulolytic bacteria even after the growth ceases because of the continuous activity of extracellular cellulase enzymes (Desvaux et al. 2000). It was observed that after 120 h, reducing sugar concentration in DSM122 cellulose medium increased by 25%. However, sugar concentration in SB, BP, and RH medium decreased by 12.6, 34.75, and 27.17%, respectively. *f.*RS value of the SB medium was observed to be close to unity (0.91), indicating that uptake rates of reducing sugar were similar to the released concentration. Whereas *f.*RS values of RH and BP medium were low, indicating sustenance of the organism in the available sugars. Extending the incubation period (up to 168 h) and/or sub-culturing further for several series did not increase the growth nor sugar concentration (data not shown) in any of the LCS media. Low growth of organism in all media resulted in low substrate conversion eventually with lower reducing sugar yield. Recovered residual biomass of different LCS media and its ethanol concentration is represented in Table 3.

**Table 3:** Comparative results of two experimental setups

	Different media	Maximum O.D.^a^	*f*.RS^b^	Ethanol (g/L)	Residual recovery (%)
Experimental setup I	SB	0.415 ± 0.017	0.91	0.58	81.6 ± 3.1
	BP	0.465 ± 0.032	0.65	0.3	82 ± 2.4
	RH	0.518 ± 0.034	0.73	0.42	84.4 ± 3.3
Experimental setup II	SAE4-SB	0.794 ± 0.044	1.18	1.21	36 ± 2.3
	SAE4-BP	0.543 ± 0.026	1.01	0.42	74 ± 3.0
	PAE4-RH	0.786 ± 0.028	1.38	1	48 ± 2.2
Standard media	DSM122 cellulose	0.691 ± 0.022	1.25	1.14	35.7 ± 1.1
	DSM122 cellobiose	0.956 ± 0.030	0.5	0.54	–

Maximum OD as achieved for the batch

^a^Values are ±SE (*n* = 3)

^b^ *f*.RS < 1 indicates that sugar consumption exceeded sugar release due to hydrolysis; *f*.RS > 1 indicates that sugar release due to hydrolysis exceeded sugar consumption; and *f*.RS = 1 indicates that sugar released due to hydrolysis was equally consumed

### Experimental setup II: serial acclimation experiment

It was observed that an actively growing culture struggled to grow when inoculated into LCS media (experimental setup I studies). Hence, in experimental setup II, residence interaction time of the microbe to the substrate was increased from 96 to 288 h, by gradually sub-culturing it from least concentration of LCS with 2.5 (grams equivalent to cellulose) to 7.5 g/L (gram equivalent to cellulose) with cellobiose as an adjunct. SAE1, SAE2, and SAE3 trials exhibited an early initiation of growth irrespective of the type of substrate, and these batches had maximum OD_600_ > 0.9. This rapid growth was due to the presence cellobiose, since it is a preferred assimilating disaccharide and a growth inducer for *C. thermocellum* than glucose (Das et al. 2013; Dionisi et al. 2014).

Even though the SAE1, SAE2, and SAE3 batches showed better growth, there was a dip in reducing sugar concentration from 0th to 120th hours across all substrates (Table 4). This could be attributed to the organism utilising the available cellobiose for growth rather than the subjected LCSs. As the residence time increased across batches, an increment in the *f*.RS values was observed. Despite the absence the cellobiose adjunct in the SAE4 batches, SAE4-SB medium had 20.4% increase in reducing sugar concentration with 672 ± 10 mg/g of cellulose equivalent released from the pre-treated substrate. RH was observed to have an 18.7% increase in reducing sugars with 636 ± 15 mg/g of cellulose equivalent being released. The corresponding *f*.RS were >1 indicating the release of sugar upon hydrolysis. Contradictorily in BP medium, *f*.RS was ~1.0 with reducing sugar of 5.13 ± 14 mg/g of cellulose equivalent. From the FTIR results, it was evident that less functional groups of LCS were disrupted, which in turn directly affected the release of reducing sugars with only 1.1% of increment from the 0th hour. Hence, the authors are of the opinion that mild alkali pre-treatment of BP may not be an appropriate choice of pre-treatment due to scanty growth of organism and little LCS conversion. The mono-sugars that were detected in SAE4-SB broth were arabinose, galactose, and cellobiose, and in SAE4-RH broth, glucose, xylose, mannose, fructose, and galactose were identified. In SAE4-BP, only galactose and cellobiose was identified.

**Table 4:** *f*.RS values of experimental setup II. *f*.RS were calculated at every 24 h interval for BP, SB, and RH

Hours	*f*.RS of BP				*f*.RS of SB				*f*.RS of RH			
	SAE1	SAE2	SAE3	SAE4	SAE1	SAE2	SAE3	SAE4	SAE1	SAE2	SAE3	SAE4
0	1.0	1.0	1.0	1.0	1.0	1.0	1.0	1.0	1.0	1.0	1.0	1.0
24	0.94	0.95	0.90	1.03	0.98	0.99	0.99	0.95	0.96	0.91	0.88	1.03
48	0.83	0.82	0.83	1.03	0.95	0.91	0.95	0.88	0.94	0.81	0.85	1.09
72	0.71	0.78	0.71	1.08	0.82	0.88	0.89	0.97	0.81	0.83	0.81	1.30
96	0.51	0.64	0.75	1.04	0.75	0.76	0.85	1.08	0.73	0.79	0.85	1.20
120	–	–	–	1.01	–	–	–	1.18	–	–	–	1.38

A two-fold increase in ethanol concentration was observed in SAE4-SB and SAE4-RH with 1.21 and 1.0 g/L, respectively, when compared to experimental setup 1. Previous reports have claimed a maximum ethanol yield of 1.4, 0.83, 0.77, and 0.53 g/L from various leafy substrates of Jamun (*Syzygium cumini*), bamboo, wild grass, and eucalyptus, respectively, using recombinant *C. thermocellum* cellulases (Mutreja et al. 2011). The present work claims an enhanced hydrolytic action of wild strain on SB and RH substrates with improved ethanol production without any genetic modification or externally added enzymes.

Consistent results were produced when inoculated into three consecutive batches having SAE4 composition. Here, the organism on acclimation became competent with a better hydrolytic capability and flourished in pre-treated LCS media without any growth enhancers. Simultaneous synergistic action of multi-enzyme complexes targeting a particular site of interest (Zverlov and Schwarz 2008) results in the formation of cellulose-enzyme-complexes (Mayer et al. 1987). Externally added enzyme-hydrolysis studies have been reported to get inhibited by its own enzyme products. Such inhibition is not exhibited in the case of synergistically acting multi-enzyme complexes (Lu et al. 2006), which could likely be a contributing reason for increased yield of reducing sugar in this experimental setup.

## Conclusion

Microbial hydrolysis is a mild and eco-friendly and cost-effective approach for hydrolysis. The pure strain of *Clostridium thermocellum* was successfully acclimated by serially incubating in increasing concentrations of respective LCSs (gram equivalent cellulose) with cellobiose as growth inducer. The acclimated organism could then uninhibitedly grow in respective LCSs without any addition of the growth inducer and also showed ethanolgenesis comparable to the standard cellulose substrate. This study clearly establishes the need for acclimating *Clostridium thermocellum* to any LCS prior to fermentation. The acclimated organism, however, showed restraint in growing in BP medium likely due to the presence of undegraded lignin. The obtained results offer an alternative avenue for cost-effective and cleaner hydrolysis process prior to fermentation. Increasing the lignocellulosic load for the whole cell hydrolytic activity may increase the total reducing sugar pool which can lessen the dependency on techniques/instruments needed to concentrate the same for ethanol fermentation. The microbial LCS hydrolysate thus obtained from agricultural waste residues can be a potential resource for biofuel. This slurry can also be fermented with other ethanolgenic organisms for enhanced ethanol titre without any added nutritional supplements.

## Abbreviations

LCS: Lignocellulosic substrate
BP: Banana pseudostem
RH: Rice husk
SB: Sugarcane bagasse
*f*.RS: Fraction of reducing sugar
